# Transfusion Strategy: Impact of Haemodynamics and the Challenge of Haemodilution

**DOI:** 10.1155/2014/627141

**Published:** 2014-08-06

**Authors:** Carl-Johan Jakobsen

**Affiliations:** Department of Anaesthesiology and Intensive Care, Aarhus University Hospital, 8200 Aarhus N, Denmark

## Abstract

Blood transfusion is associated with increased morbidity and mortality and numerous reports have emphasised the need for reduction. Following this there is increased attention to the concept of patient blood management. However, bleeding is relatively common following cardiac surgery and is further enhanced by the continued antiplatelet therapy policy. Another important issue is that cardiopulmonary bypass leads to haemodilution and a potential blood loss. The basic role of blood is oxygen transport to the organs. The determining factors of oxygen delivery are cardiac output, haemoglobin, and saturation. If oxygen delivery/consumption is out of balance, the compensation mechanisms are simple, as a decrease in one factor results in an increase in one or two other factors. Patients with coexisting cardiac diseases may be of particular risk, but studies indicate that patients with coexisting cardiac diseases tolerate moderate anaemia and may even benefit from a restrictive transfusion regimen. Further it has been shown that patients with reduced left ventricular function are able to compensate with increased cardiac output in response to bleeding and haemodilution if normovolaemia is maintained. In conclusion the evidence supports that each institution establishes its own patient blood management strategy to both conserve blood products and maximise outcome.

## 1. Status in Frequency of Transfusion and Impact on Outcome in Cardiac Surgery

The concept of patient blood management is gaining increased attention. During the last decade numerous reports have emphasised the need for reductions in transfusions of blood and blood products as allogeneic red blood cell (RBC) transfusions are associated with increased morbidity and mortality [1–10], increased risk of severe postoperative infections [5], adverse effects or risk of transferring pathogens [11, 12], relatively high costs, and shortage of blood bank products [6, 12–14]. Postoperative severe bleeding is relatively common following cardiac surgery compared to other surgical specialties and is considered a serious complication associated with increased morbidity and mortality [14–21]. Within the cardiac surgery population, patients with advanced age and long cardiopulmonary bypass (CPB) are especially at risk of postoperative bleeding [18, 19]. Moreover, it is well known that excessive bleeding may be caused by surgical factors and impaired haemostasis due to enhanced fibrinolysis, platelet dysfunction, haemodilution, acidosis, hypothermia, and consumption of coagulation factors as well as the surgical trauma alone [22, 23].

**Figure 1:**
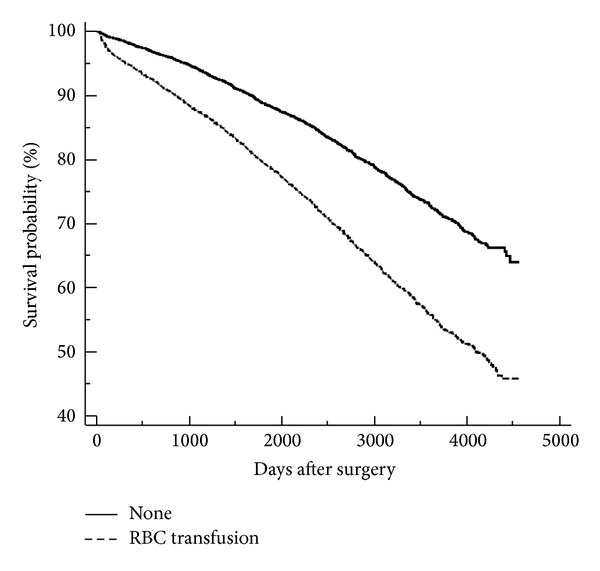
Unadjusted long term survival following standard cardiac surgery procedures (CABG, AVR, and MVR) divided on perioperative blood transfusion [24]. Patients dying within first 30 days postoperatively were excluded from analysis.

During recent years several cardiac surgery studies have reported on the long term mortality after transfusion with blood and blood products [6–10]. Although not all [7], the majority of studies report a higher long term mortality after blood transfusion [6, 9, 10, 24] (Figure 1). However, the reports are mainly from designated types of surgery [7–9] or single centre studies [6, 8, 9]. In some studies the Kaplan-Meyer plots tend to run parallel after the first month when including the immediate postoperative mortality, indicating less impact on the long term survival [6, 9, 10].

One of the last published evidence based guidelines for the transfusion of RBC concluded that “red blood cell transfusions should not be dictated by a single haemoglobin (Hb) “transfusion trigger”, but instead should be based on the patient's risk of developing complications of inadequate oxygenation.” Furthermore, that RBC transfusion would generally be indicated, but not mandatory, at Hb levels lower than 6.0 g/dL (3.7 mmol/L) and rarely indicated in patients with haemoglobin higher than 10.0 g/dL (6.1 mmol/L) [25]. In many types of major surgery these recommendations have been followed and the use of transfusions reduced. However, in cardiac and vascular surgery the use of blood and blood products is still common clinical practice.

**Figure 2:**
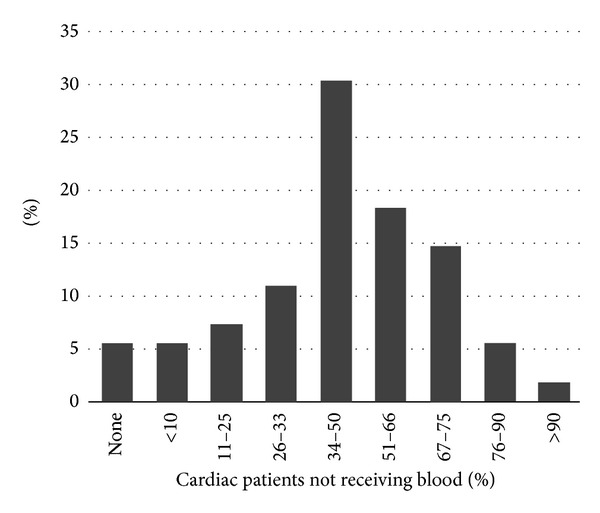
The fraction of patient not receiving perioperative blood transfusion in European cardiac centers (survey from 2005: 119 European institutions covering 117,800 cardiac procedures).

From a survey of cardiac procedures covering monitoring, anaesthesia and transfusions carried out in 2005, it was found that almost 40% of European institutions used blood or blood products in more than half of their cardiac surgery patients [26]. When calculating the number of surgical procedures in each institution and number of patients not receiving blood or blood products, 55.7% of European cardiac surgery patients received blood or blood products in the perioperative period. However, the difference in transfusions from less than 10% of patients to 100% that do receive blood or blood product transfusion (Figure 2) is interesting and indicates that transfusions are guided by local policies and not evidence based practice.

## 2. Continued Antiplatelet Therapy

Patients referred for coronary artery bypass graft (CABG) are more commonly treated with antiplatelet agents especially aspirin and oral adenosine diphosphate (ADP) receptor antagonists together with newer alternative low molecular heparin drugs, like fondaparinux. Long term aspirin therapy is the standard of care in patients with coronary artery disease, while concomitant treatment with oral ADP receptor antagonists is recommended for patients with recent acute coronary syndrome (ACS) or percutaneous coronary intervention (PCI) [27–29]. Studies do not agree on the impact of these drugs on perioperative bleeding. Studies have found both reduced mortality and no difference in bleeding [30] or no difference in bleeding complications in patients treated with antiplatelet drugs [31, 32]. This is in contrast to other studies reporting higher transfusion requirements without effect on mortality [33] or increased bleeding and transfusion as well as increased risk of myocardial infarction [34].

In the CRUSADE study following established guidelines a significant increase in transfusion requirements in patients continuing on clopidogrel was observed compared to patients where the drug was discontinued more than five days before surgery [35]. Similarly, large cohort studies found significant increases in major bleeding and reoperation rates in clopidogrel-exposed CABG patients [33, 36].

The use of antiplatelet agents at the time of CABG carries both benefits and risks. The drugs are effective in reducing ischemic events in high-risk patients awaiting surgery. After surgery drugs may prevent graft occlusion and recurrence of ischemic episodes. However, antiplatelet drugs also aggravate perioperative bleeding followed by increased perioperative blood loss, increased transfusion requirements, and hemodynamic instability [35, 37, 38]. As the indications for oral antiplatelet drugs expand, cardiac surgeons frequently have to make decision about the timing of surgery in patients exposed to these drugs. Current international guidelines recommend discontinuing aspirin two to ten days before elective cardiac surgery, while the ADP receptor antagonist clopidogrel should be withdrawn at least five days before elective CABG [39, 40].

## 3. Circulation and Red Blood Cells (Haemoglobin)

### 3.1. Haemoglobin and Organ Oxygenation

**Figure 3:**
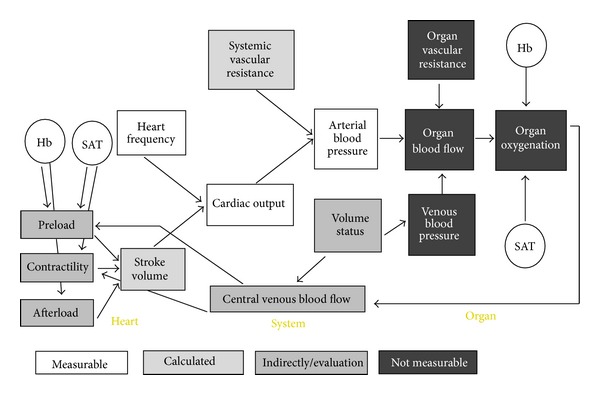
Variables and factors influencing and determining organ oxygenation and the measurement possibilities.

The basic role of blood is to transport and deliver oxygen to the organs to maintain organ function. The determining factors and variables influencing organ oxygenation are shown in Figure 3. It is evident that clinical and some hemodynamic measurements are imperative to gain the required information for decision making. The question is how comprehensive monitoring should be in order to fulfill these needs. Even with the most extensive invasive haemodynamic monitoring the obtained information is not fully adequate, as individual organ monitoring is not possible or available in clinical practice. We do have some intervention possibilities for blood pressure, heart rate, haemoglobin level, and volume status and it is possible to operate influence factors such as oxygen saturation, vascular resistance, preload, and cardiac output, while organ blood flow and organ oxygenation are difficult to influence directly.

### 3.2. Circulation, Oxygen Consumption, and Haemoglobin

**Figure 4:**
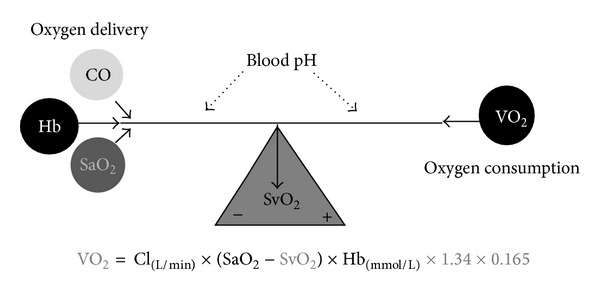
Factor and relations between the oxygen delivery and oxygen consumption.

The correlation between blood flow, oxygen delivery, and consumption together with haemoglobin can easily be visualised (Figure 4). The figure demonstrates that oxygen delivery in principle is determined by only three variables, cardiac output (CO), haemoglobin (Hb) level, and oxygenation (SaO_2_). Additionally, blood pH value influences the binding of oxygen to haemoglobin.

If oxygen delivery is out of balance with oxygen consumption, the compensation mechanisms are relatively simple, as a decrease in haemoglobin must result in a compensatory increase in either cardiac index or oxygen extraction or a combination. Furthermore, the relation is independent of the fact that CO changes with age [41] and that the ratio of CO to total blood volume changes from roughly 3 : 1 in small children to 2 : 3 or lower in the elderly cardiac patient.

**Figure 5:**
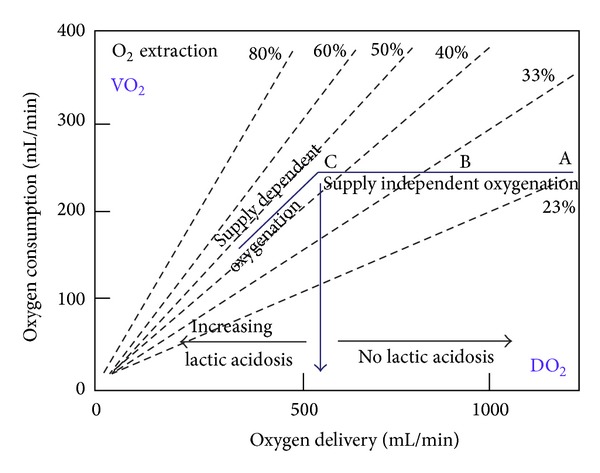
Oxygen consumption dependency of oxygen supply and influence of oxygen extraction fraction.

A valuable parameter in evaluating need for inotropic support or RBC transfusion is whether oxygen consumption is adequately covered by oxygen delivery. In general, oxygen delivery to the cells is higher than actual consumption. When oxygen consumption is high (i.e., during exercise) the increased oxygen requirement is usually provided by an increased cardiac output. However, low cardiac output, low haemoglobin concentration (anaemia), or low haemoglobin O_2_ saturation will result in an inadequate delivery of oxygen, unless a compensatory change occurs in one of the other factors. Alternatively, if oxygen delivery falls relative to oxygen consumption, the tissues extract more oxygen from the haemoglobin and the saturation of mixed venous blood (SvO_2_) falls below 70% (A-B in Figure 5). A reduction below point “C” in Figure 4 cannot be compensated by an increased oxygen extraction and results in anaerobic metabolism and lactic acidosis, and oxygen consumption becomes totally dependent on oxygen supply.

Oxygen is carried in the blood in two forms: primarily by haemoglobin but a very small amount is dissolved in the plasma. When fully oxygen saturated (PO_2_ > 13.3 kPa) only 3 mL of oxygen will be dissolved in every litre of plasma. If the PO_2_ of oxygen in arterial blood (PaO_2_) is increased significantly (breathing 100% oxygen), a small amount of extra oxygen will dissolve in the plasma at a rate of 0.023 mL O_2_/100 mL blood/kPa PO_2_. Normally, there will be no significant increase in the amount carried by haemoglobin if oxygen saturation is already higher than 95%.

In the literature there is some indications that patients with coexisting cardiac diseases may have a particular risk of developing impaired oxygenation at lower haemoglobin levels, but the documentation for this is not that convincing. In patients undergoing cardiac surgery the relation between transfusion and postoperative morbidity and mortality is more uncertain. Studies of cardiac surgery patients have shown increased mortality after RBC transfusions [42, 43]. Further, others have found that lowering of the Hb threshold for transfusion in patients undergoing coronary artery bypass surgery (CABG) from 9 to 8 g/dL (5.5 to 5.0 mmol/L) was neither followed by a higher mortality nor followed by an increase in adverse effects by accepting the lower postoperative haemoglobin [44]. This indicates that patients with coexisting cardiac diseases, including coronary artery disease (CAD), tolerate moderate anaemia. Another study showed that patients may even benefit from a restrictive transfusion regimen followed by a lower morbidity and mortality [2]. The mean haemoglobin at transfusion in this study was 8.4 g/dL (5.1 mmol/L) and both ICU and overall mortality rate were higher in transfused patients. This was confirmed in multivariate analyses and a matched pair analysis [2]. The findings were further supported in an ICU study where patients with known cardiovascular disease had equal level of mortality and less organ dysfunction if in a transfusion restrictive group [45].

In contrast, one study indicated that RBC transfusions may decrease mortality in elderly patients with acute myocardial infarction and an admission haematocrit below 30% [46]. Another study shows increased in-hospital mortality in patients with lower preoperative haemoglobin levels [47]. However, the first study is not without methodological challenges and needs careful analysis as patients with normal haemoglobin levels seemed to be treated more intensely. Previously, we have demonstrated a correlation between preoperative haematocrit (Hct) and 30-day mortality [26]. However, when compensated for EuroSCORE [48–50] the 30-day mortality was independent of haemoglobin level and only correlated with the EuroSCORE. The data showed a strong relationship between actual RBC transfusion and residual EuroSCORE (EuroSCORE minus age factor) and interestingly also the lack of correlation between age higher than 74 years and RBC transfusion (Table 1).

The first priority of perioperative fluid therapy in surgical patients is to achieve optimal filling of the heart with crystalloids and colloids to optimise cardiovascular function, decrease postoperative morbidity, and shorten the length of hospitalisation [51, 52]. In cardiac surgery patients and many intensive care patients with PaO_2_ > 95%, optimisation of arterial saturation only adds little to oxygen delivery, leaving only increasing CO or Hb as possible means to increase oxygen delivery. Infusion of colloids reduces the Hb level and thus, despite unchanged total haemoglobin content, oxygen delivery in theory. However, the impact on CO normally compensates for that and in hypovolaemic patients especially the overall impact may be substantially higher CO and thus oxygen delivery. Following infusion of RBC in this type of patients may have a double effect on oxygen delivery as both Hb and CO are increased. In contrast, in severely overloaded patients where further compensation in cardiac output is compromised or even deteriorated after infusion of colloid or RBC, there might be no effect on the oxygen delivery. In these cases inotropic support may be the only way to increase oxygen delivery.

Many cardiac or critically ill patients are not monitored with pulmonary artery catheters and hence information of CO and SvO_2_ is not available. Central venous oxygen saturation (ScvO_2_) obtained from the superior vena cava has been proposed as a surrogate for SvO_2_ and may reflect the balance between oxygen supply and demand. Studies have shown that the difference between ScvO_2_ and SvO_2_ is consistently about 5% across a wide range of cardiorespiratory conditions in both animals and humans [53, 54]. However, ScvO_2_ obtained from a central venous catheter is not fully interchangeable with mixed SvO_2_, but it can give some indication of oxygen balance in patients with peripheral saturation, CO and Hct within normal limits [55].

### 3.3. The Haemodilution Challenge

**Figure 6:**
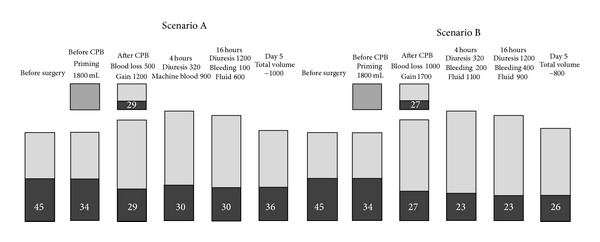
Perioperative changes in haematocrit and total haemoglobin. SCENARIO A: optimal perioperative management; impact of accurate blood conservation, use of cell-saver/retransfusion of machine blood and autotransfusion. SCENARIO B: nonoptimal perioperative handling; no use of cell-saver/retransfusion of machine blood and postoperative autotransfusion.

One of the principal differences in cardiac surgery compared to other types of major surgery is the use of cardiopulmonary bypass (CPB). This leads to a 20–30% haemodilution and when weaning from CPB a relatively big portion of red blood cells (RBC) are left in the heart lung machine (HLM) (Figure 6). Although the surgeon is very careful during the procedure, a smaller portion of blood is always lost. Using either cell-saver or retransfusing of suctioned blood will, however, limit the total loss. If using autotransfusion in the postoperative phase the perioperative blood loss during cardiac surgery can be diminished to approximately one unit of RBC (SCENARIO A) and there is thus no need for transfusion. However, if none of these procedures are used, the overall result will be a considerable blood loss and a low postoperative haematocrit level (SCENARIO B). Consequently, the patient will inevitably receive 1–3 units of RBC. If the patient continuously has problems with bleeding or haemodynamic instability, the risk of adding plasma or platelets is reasonably high.

Some studies have found low haemoglobin levels during CPB to be associated with increased postoperative morbidity and mortality [56, 57] while other studies have not been able to find such a correlation [43, 58].

Patients with coronary artery disease (CAD) may have an increased risk of myocardial ischemia in combination with low haemoglobin levels. However, experimental and clinical data indicate that persons with CAD tolerate moderate normovolaemic haemodilution well [59–62]. Interestingly, data from our database show that patients with CABG received less blood and blood products (Table 2).

Regional changes in myocardial systolic and diastolic contractile dysfunction [63, 64] and ECG signs of myocardial ischemia [65–68] may develop in areas supplied by a compromised coronary artery at low haemoglobin levels. Additionally, haemoglobin levels below 6.0 g/dL (3.7 mmol/L) have been associated with increased postoperative mortality in patients with coexisting cardiovascular disease, including CAD [69].

During both haemodilution [59] and blood transfusion [70] patients with a left ventricular ejection fraction between 25% and 85% responded similarly. This indicates that also patients with a reduced left ventricular ejection fraction are able to compensate with increased cardiac output in response to haemodilution if normovolaemia is maintained. However, the number of patients with low ejection fractions was limited and the findings should not necessarily be extrapolated to the low range of patients with left ventricular ejection fraction.

Patients above 65 years without known cardiac disease tolerated haemodilution to a haemoglobin of 8.8 g/dL (5.4 mmol/L) well [71]. Beta-blocked CAD patients between 35 and 81 years responded adequately during haemodilution with an increase in cardiac output and oxygen extraction [59]. Similarly, during blood transfusion the compensatory changes in cardiac output, oxygen delivery, and oxygen consumption seemed to be independent of age (32 to 81 years) [70]. However, this study had relatively few patients above 75 years, stressing that these findings may not apply to the very old patients.

Patients with significant mitral insufficiency, including those with atrial fibrillation, tolerate moderate haemodilution to a haemoglobin level of 10 g/dL (6.1 mmol/L) [72]. Tolerance of acute haemodilution or anaemia in patients with other valve abnormalities is less well known. Theoretically, patients with aortic and pulmonary stenosis may be less tolerant to haemodilution as the increase in cardiac output during haemodilution may be limited due to the valvular stenosis. A more liberal transfusion regimen thus appears indicated in such patients. However, in the postoperative phase these patients may most likely be treated as patients without coexisting cardiovascular diseases.

In patients with coexisting cardiovascular diseases refusing RBC transfusions for religious reasons, postoperative haemoglobin levels below 6.0 g/dL (3.7 mmol/L) were associated with increased mortality and morbidity and an increasingly greater difference in mortality and morbidity between patients with and without coexisting cardiovascular diseases [69].

In order to avoid or minimize transfusions in patients with low haemoglobin it should be considered whether preoperative haemoglobin adjustment is needed. However, this is not possible in acute or life threatening situations. In this evaluation it is evident that the 20–30% haemodilution during CPB might be the primary concern if the patient does not have cardiac capacity to tolerate very low post-CPB haemoglobin or that a needed high HTLM cardiac output during CPB may increase perioperative complications. The overall conclusion is that even patients with coexisting cardiac diseases tolerate moderate haemodilution or acute anaemia well if normovolaemia is maintained. However, an excessive and aggressive haemodilution may cause myocardial ischemia which is reversible with a blood transfusion, even in normovolaemic patients [64, 68, 73], and the lowest acceptable haemoglobin in patients with coexisting cardiovascular diseases is approximately 6.0 g/dL (3.7 mmol/L, haematocrit 0.18–0.20) [69]. Patients where pre-CPB calculations indicate a lower level during or right after CPB might be the target for preoperative haemoglobin adjustment with erythropoietin with or without autologous blood transfusion.

### 3.4. Haemodynamic Effect of Relative Anaemia

The haemoglobin level at which regional cardiac ischemia may occur varies considerably and depends on both the degree of coronary stenosis [74] and whether it is a single- or a multivessel CAD [75]. Patients with or at risk of CAD should thus not automatically receive a transfusion at a specific haemoglobin level but only if oxygenation is inadequate as suggested by the American Society of Anaesthesiologists (ASA) [12]. The question is whether anaemia-related myocardial ischemia is reversible by blood transfusion, which seems to be the case in some experimental [64] and clinical [68, 73] settings.

Patients with progressively lower haemoglobin levels also have other risk factors such as diabetes, preoperative congestive heart failure, prior coronary artery bypass operation, low left ventricular ejection fraction, and a higher frequency of emergency surgery [56]. A high correlation has been found between such risk factors and a low haemoglobin level [58] and the independent contribution of a low perioperative haemoglobin level to mortality is difficult to assess. After adjusting for other risk factors, only a haematocrit below or equal to 14% during CPB remained an independent predictor for increased mortality [57].

Only a prospective randomised study design can definitively determine whether varying haemoglobin transfusion triggers affect morbidity and mortality. In a study of ICU patients it was demonstrated that a haemoglobin transfusion trigger of 7 g/dL (4.3 mmol/L) did not negatively impact on mortality and morbidity, neither in general ICU patients nor in patients with coexisting cardiac diseases [1, 45].

Several studies have shown the importance of controlling haemodynamics during cardiac [76, 77] as well as noncardiac surgery [78]. Haemodynamic management is generally less strict postoperatively compared with intraoperatively. Therefore, episodes of tachycardia may occur, which may be associated with ST-segment depression, in particular in patients with *t* low haemoglobin levels [67]. Treating tachycardia alleviates ST-segment depression [68] and thus postoperative heart rate control. Preventing tachycardia and following ST-segment depression is thus imperative [79] in patients with or at risk of CAD. In addition, postoperative oxygenation is generally lower than during peroperative mechanical ventilation with a high inspiratory oxygen concentration. Therefore, higher postoperative haemoglobin levels of 7 to 8 g/dL (4.3–4.9 mmol/L) may be justified in patients with coexisting cardiac diseases.

The question of when transfusion is appropriate in a patient with a coexisting cardiac disease thus remains unanswered. At a haemoglobin level lower than 6.0 g/dL (3.7 mmol/L) blood transfusions may be indicated in most patients and in particular in patients with coexisting cardiac diseases [25, 69, 80, 81]. Furthermore, it has been shown in cardiac surgery patients that oxygen consumption only increased following a blood transfusion when oxygen consumption was very low before blood transfusion [70]. As this remains impossible to determine also in patients with coexisting cardiac diseases, the haemoglobin level, at which a transfusion would generally be indicated, should follow the guidelines from the ASA, stating that “red blood cell transfusions should be based on the patient's risk of developing complications of inadequate oxygenation” [25]. The immediate question is then, what are the signs of a beginning inadequate oxygenation in patients with coexisting cardiac diseases? Inadequate oxygenation may become globally manifested in the form of general haemodynamic instability with a tendency to hypotension and tachycardia despite normovolaemia, oxygen extraction higher than 50% [80–82], or myocardial ischemia detected by continuous 5-lead ECG monitoring, ideally with automatic ST-segment analysis [65, 68, 83] and by new wall motion abnormalities in transoesophageal echocardiography [84]. ST-segment depressions above 0.1 mV or new ST-segment elevations above 0.2 mV during more than one minute generally are regarded as a marker of myocardial ischemia. During progressive haemodilution, primarily ST-segment depression is observed [68], suggesting subendocardial ischaemia. Such anaemia-related ischaemia may, in case of tachycardia, be reversed by decreasing the heart rate [68] and by increasing the haemoglobin level 1-2 g/dL (0.6–1.2 mmol/L) by transfusion [64]. New wall motion abnormalities clinically detected by transoesophageal echocardiography may be the result of myocardial ischemia and can be treated by a minor increase in haemoglobin of 1 to 2 g/dL (0.6–1.2 mmol/L) [64].

Early signs of inadequate circulation are general haemodynamic instability characterised by relative tachycardia and hypotension [81, 85] together with an oxygen extraction fraction above 50%, a low mixed venous oxygen partial pressure (PvO_2_), and a decrease in oxygen consumption [80, 81]. An oxygen extraction higher than 50% has been found to indicate exhaustion of compensatory mechanisms in several studies [86–89] and might thus represent a transfusion indication. Oxygen consumption decreases very late and at very low haemoglobin levels during progressive normovolaemic haemodilution [89, 90] in conditions where oxygen extraction has increased and PvO_2_ has already decreased [88]. Therefore, any decrease in oxygen consumption of more than 10% at low haemoglobin levels [71] should be viewed as a potential sign of a compromised oxygenation and a blood transfusion should be considered if normovolaemia has been achieved. In patients with coexisting cardiac diseases, the principles of RBC transfusion are not considerably different from healthy patients and must also largely be based on early signs of impaired oxygenation of specific organs or the entire organism. Impaired oxygenation may be reached at higher haemoglobin values than in healthy patients and most patients have considerable variations in haemodynamic values.

**Figure 7:**
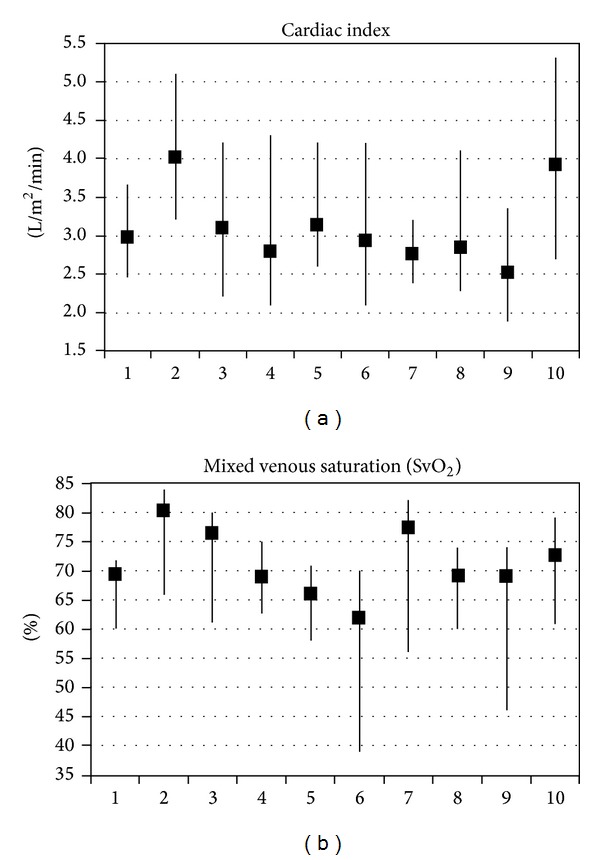
Variances in cardiac index and SvO_2_ in elective patients the night before surgery [91].

A previous study found considerable interpatient differences and intrapatient variation in patients monitored the night before cardiac surgery (Figure 7) [91]. The most pronounced intrapatient variation was found in the cardiac index, ranging from 1.9 to 5.3 l/min/m^2^. Most of the patients had periodic SpO_2_ values ≤92 and half of them in more than 15% of the observations. Overall a SvO_2_ < 70% was found in more than 40% of observations and less than 64% in more than 20% of the observations together with drops below 50% without obvious reasons. Although the number of patients in the study was low the conclusion was that intrapatient variation was unexpectedly high in most hemodynamic variables. This demonstrated the challenge in using hemodynamic parameters to guide treatment and indicated that goal oriented therapy using currently accepted values may result in overtreatment in some patients [91].

## 4. The Function of Stored Red Blood Cells

A restrictive RBC transfusion policy has proven equally effective and possibly even superior to a more liberal transfusion strategy [1]. Although there currently is no clear explanation for this effect, infections [92], immunosuppression [3], and the age of the RBC at the time of transfusion [5, 93, 94] have been suggested as possible impact factors.

Storage lesions are morphological and biochemical alterations found after RBC storage [95]. The clinical consequence of the storage lesions is a reduced survival time of red blood cells after the transfusion. Whether stored RBC is also compromised in transporting oxygen to tissue is still controversial. Several clinical [93, 96, 97] and experimental animal studies [98, 99] support the notion that RBC older than 3 weeks have a reduced ability to transport oxygen to the tissues. Animal studies have found a malperfused and underoxygenated microvasculature after haemodilution [100] and a 26% decrease in micro vascular oxygen concentration after transfusion with washed human RBCs in haemodiluted rats [101]. In human a recent study has shown increased risk of severe postoperative infections after cardiac surgery associated with the use of old RBC [5].

However, the findings are not uniform as a recent clinical study could not demonstrate an effect of stored blood on oxygenation parameters [102] and another did not observe an increase in morbidity in coronary artery bypass graft surgery patients receiving old RBCs [14] or could not find a correlation between median ages of the RBC units and clinical outcomes [103].

These differences in study outcomes may be explained by the inherent difficulty to measure tissue oxygen concentrations in the clinical setting. To estimate the effect of RBC transfusion on tissue oxygenation in patients, indirect measuring techniques have been used as surrogate endpoints. In addition, the diversity in the design of the performed studies complicates a proper comparison of their results. There seems to be an agreement that when a substantial part of the units issued for transfusion is stored for more than 3 weeks, a discussion about fresh and stored RBC could be clinically relevant [104].

## 5. Conclusions and Recommendations

The evidence supports that each institution establishes its own patient blood management strategy to both conserve blood products and maximise outcome.

Newer transfusion guidelines in patients with coexisting cardiac diseases are similar to those in patients without such comorbidities. Thus, RBC transfusions are most often indicated at haemoglobin levels below 6.0 g/dL (3.7 mmol/L) and hardly ever at haemoglobin levels above 10 g/dL (6.1 mmol/L) [25]. In cardiac surgery, especially in CAD patients, the lower limit should probably be elevated to 7 g/dL (4.3 mmol/L), though no available hard evidence. Institutions should evaluate how they approach transfusion and each physician should evaluate each patient for signs and symptoms of ischemia or imbalance in oxygen supply and delivery, where the major challenge is how to decide when that imbalance is occurring in certain tissues. Evidently such signs may be reached at higher haemoglobin values than in healthy patients, but further studies are needed to fully confirm that transfusing these patients at a higher haemoglobin level positively is making more good than harm.

Following this teams and institutions need to conduct open discussions about how they will approach patient blood management and instead of previous fixed haemoglobin levels the best practice would be individual haemoglobin/haematocrit triggers.

## Transfusion Strategy: Issues to Consider When Making a Strategy

1. Strategy of transfusion: Make local policy and guidelines for transfusion including
  - Preferably developing an algorithm for transfusion
  - Education and knowledge of physicians and nurses
  - Policy on blood saving precautions, mechanical or medical
  - Evaluation of cost of blood/blood products and cost alternatives
  - Availability of blood and blood products
  - Transfusion of only actual bleeding and/or unstable patients
  - Safety related to need
  - Affection of immune system acceptable?
2. Preoperative evaluation
  - Deciding blood saving precautions
  - Cardiovascular reserve
  - Defining acceptable blood loss/haematocrit/absolute haemoglobin content.
3. Evaluate perioperative
  - Haematocrit look at it—do not treat it but
  - Evaluate the absolute amount of haemoglobin.
4. Reevaluate cardiovascular capacity and decide transfusion need.

## Figures and Tables

**Table 1:** The relation between EuroSCORE (minus age factor) and age score related to the percentage of patients receiving blood transfusions among 10,889 patients from 1999 to 2010 at Aarhus University Hospital.

EuroSCORE (minus age score)			Age score		
Score	Number	Blood %	Age	Number	Blood %
0-1	3302	23.5	<60	3002	28.2
2-3	5444	32.3	60–64	1554	32.1
4-5	2977	42.0	65–69	1887	39.3
6-7	1338	48.8	70–74	1996	39.8
8-9	744	58.6	75–79	1649	45.2
10-11	575	66.7	80–84	646	47.5
>11	516	77.9	>84	155	47.7

**Table 2:** Transfusion of blood or blood products related to cardiac surgery.

Procedure type	Number	Transfusion %
CABG	5695	38.9
Single valve	2373	39.2
CABG plus valve	1152	52.1
Single other procedure	760	45.4
Double procedures	472	55.1
Aortic surgery	437	64.5

All	10889	42.6
